# Association between the posterior part of the circle of Willis and the vertebral artery hypoplasia

**DOI:** 10.1371/journal.pone.0213226

**Published:** 2019-09-12

**Authors:** Virginija Gaigalaite, Jurate Dementaviciene, Augenijus Vilimas, Danute Kalibatiene

**Affiliations:** Faculty of Medicine, Vilnius University, Vilnius, Lithuania; Universitatsklinikum Freiburg, GERMANY

## Abstract

**Background:**

It is not clear whether the configuration of the posterior part of the circle of Willis (CW) depends on the proximal part of the vertebrobasilar system. Our aim is to evaluate the posterior part of CW in association with different size of vertebral arteries (VA) in healthy volunteers.

**Materials and methods:**

The present study was based on a sample of 923 healthy volunteers who were examined from 2013 through 2018. The duplex ultrasonographic examination of the extracranial vertebral (VA) and carotid arteries was performed. VA was defined as hypoplastic (VAH) when VA diameter in the entire course was less than 2.5 mm. All the participants underwent magnetic resonance angiography (MRA) examination. All the component vessels of the circle of Willis were assessed in each individual. We classified the posterior communicating artery (PCoA) as presence PCoA, absence/hypoplastic PCoA and fetal-type posterior circle of Willis (FCW) in which the major stem of the posterior cerebral artery (PCA) arises from ipsilateral internal carotid artery (ICA). The comparison of the posterior part of CW was made in subjects with normal VA and VAH of a different degree (communicating with basilar artery (VAH-BA) and not communicating with the basilar artery (VAH-PICA)).

**Results:**

FCW was found in 15.9% of subjects, bilaterally–in 2.3%. FCW was more frequent in individuals with VAH than in those with normal VA (accordingly, 28.8% vs. 13.5%, p<0.001. Moreover FCW was recorded in 50% of the subjects with VA—PICA in comparison with 13.5% of those with normal VA and 22.8% with VAH—BA, p<0.005. On the contrary, absence/hypoplasia of both PCoA was mostly found in the group with normal VA in comparison with VAH-BA and VAH-PICA (accordingly, 50.7%, 38.6% and 12.5%, p<0.01).

**Conclusion:**

Individuals with VAH have a different pattern of the posterior part of CW in comparison with those with normal VA. With the increasing degree of VAH, the proportion of FCW increases, while the proportion of absence/hypoplastic of both PCoA decreases.

## Introduction

**Fig 1:**
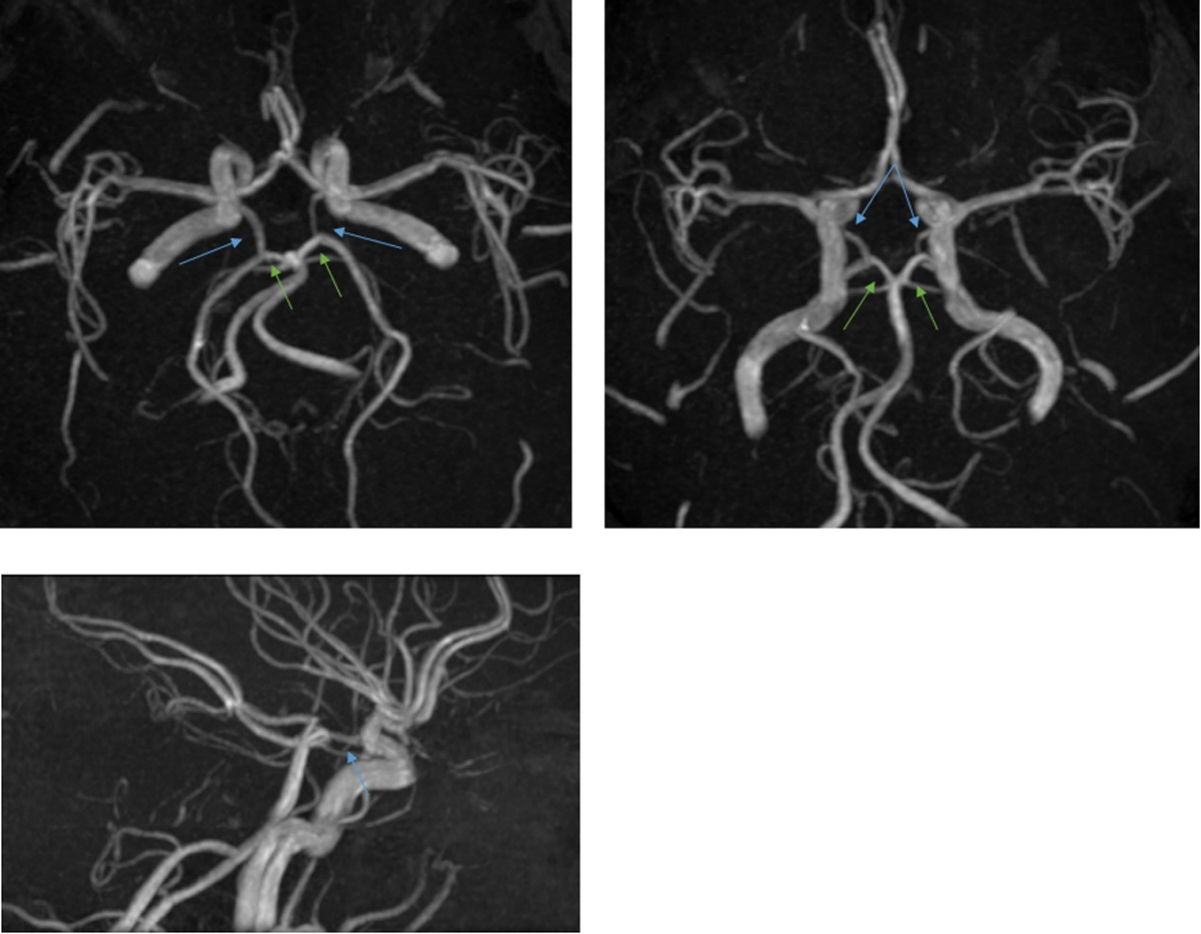
Complete posterior part of circle Willis. Blue arrows point to PCoA. Green arrows point to P1 segments of PCA.

**Fig 2:**
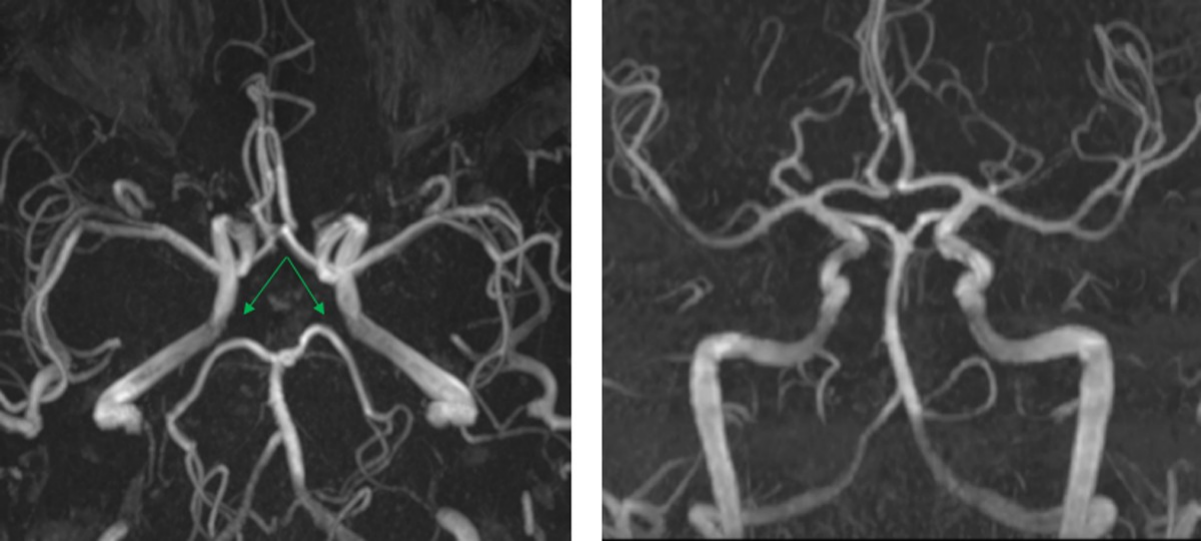
Absence of both posterior communicating arteries. Green arrows point to absence of both PCoA.

**Fig 3:**
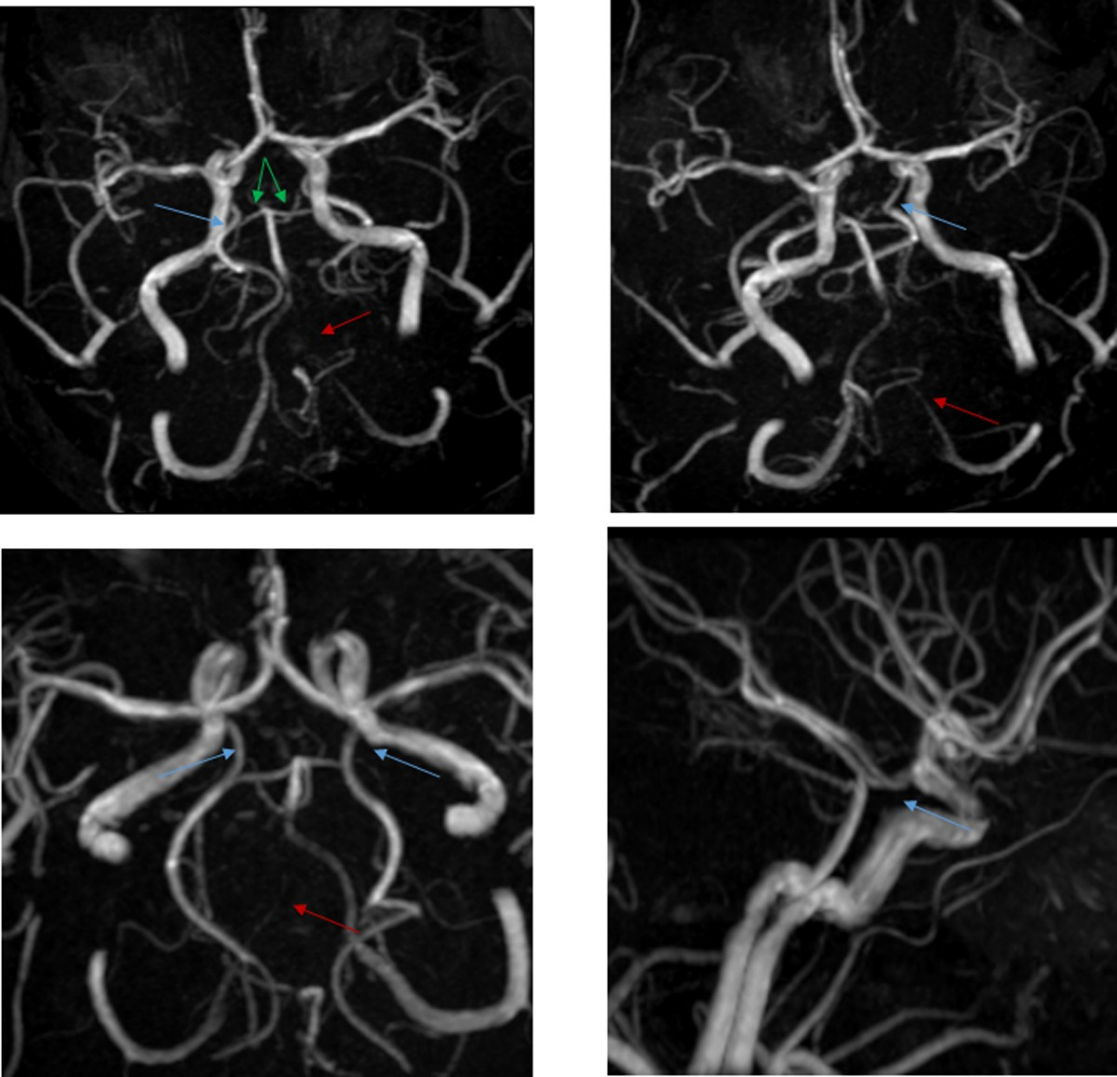
Fetal circle Willis. Blue arrow points to fetal posterior cerebral artery, green arrow points to absence of P1 segment, red arrow points to hypoplastic vertebral artery.

The Circle of Willis (CW) is a major intracranial collateral circulation that has an important role in ischemic events. The posterior collaterals are usually classified as one of the three variants: an adult configuration, a transitional configuration and a fetal configuration. The main difference between these variants is the relation between the diameter of P1 segment of the posterior cerebral artery (PCA) and the diameter of the posterior communicating artery (PCoA) [1], [2], [3]. The most common configuration of the posterior part of CW is described as adult configuration (Figs 1 and 2). In these cases, the PCA is a terminal branch of the vertebrobasilar system. The diameter of the precommunicating part (P1) of PCA is larger than the diameter of the posterior communicating artery (Pco A) connecting the vertebrobasilar and carotid systems. The presence of PCoA enables to redistribute the blood flow in both directions through PCoA in cases of diminished blood supply in the internal carotid artery (ICA) or vice versa in the vertebrobasilar system. However in more than 30% of individuals PcoA is hypoplastic or absent [3], Fig 2. In the transitional configuration which is observed in about 7% [2] of subjects the PCoA and P1 have an equal diameter. In the minority of cases the configuration of the posterior part of CW is the so called fetal-type of the posterior circle of Willis (FCW) (or fetal PCA). FCW (Fig 3) is a morphological variant of the cerebrovascular anatomy in which P1 is smaller than PCoA and PCA arises directly from the terminal ICA, with (partial fetal) or without (full fetal) an intact P1 segment connecting PCA to the basilar artery [1], [2], [3]. In this variant, the larger brain area is dependent on ICA and could be more prone to develop large ischemic strokes in cases of carotid artery stenosis or occlusion. As described by many authors [1], in these cases the collateral circulation between the anterior and posterior circulation through secondary collaterals, i.e. leptomeningeal vessels cannot develop since both, the middle cerebral artery and PCA are connected to the same internal carotid system.

As described by many authors [1], [2], [3], [4], during the embryological development, ICA is the first cerebral artery to form and it provides all the blood required by the primitive brain. As the brain stem, cerebellum and occipital lobe enlarge, the ICA supply becomes insufficient and the posterior circulation is developing. At this stage, the posterior circulation is not yet completely developed. It consists of primitive arterial networks that originate from the anterior circulation. After the development of the basilar artery (BA) and vertebral arteries (VA), these anastomoses regress. Failed regression results in anomalous connections between the anterior and posterior circulation in postnatal life. The most common form of these anastomoses is the fetal PCA. During the fetal brain development, the frequency of adult and fetal configurations increases, while the number of transitional configurations decreases [1].

Insufficient attention has been given to the FCW coexistence with other vascular congenital variants and its influence on both cerebral circulation and neurological symptomatic. It was described [5] that the coexistence of FCW, basilar artery (BA) hypoplasia and vertebral artery hypoplasia (VAH) was more common in patients with cerebral ischemia, i.e. this arterial variant may increase TIA/stroke risk. According to [6], individuals with FCW have an 18% reduction in BA diameter. It is not clear if the configuration of the posterior part of CW depends on the proximal part of the vertebrobasilar system, more exactly, on the vertebral artery (VA) diameter and in cases of a small diameter vertebrobasilar system, which configuration of posterior collateral circulation is more beneficial.

Our aim is to evaluate the posterior part of CW in association with different sizes of VA (normal diameter, VAH of different degree (communicating with the basilar artery (VAH-BA) and not communicating with the basilar artery and terminating in posterior inferior cerebellar artery (PICA), neck or aplasia (VAH-PICA)) in subjects free from stroke and TIA.

## Material and methods

The present study was based on a sample of 923 healthy volunteers. All of them were examined by magnetic resonance imaging (MRI) and magnetic resonance angiography (MRA) in the Republican Vilnius University Hospital from 2013 through 2018.

The inclusion criteria were as follows: (1) no history of transient ischemic attack, ischemic or hemorrhagic stroke; (2) no disabling neurological deficits on examination; (3) extracranial or intracranial vessels without significant stenosis (>50%) or occlusion; (4) the study excluded the patients who did not undergo MRI or MRA investigation, their intracranial vessels were not visualised or they refused to participate in the study.

### Imaging studies

All the participants underwent MRA examination using 1.5 Tesla MRI (GE Optima MR450w 1.5T MRI System) for the brain and CW evaluation.

The following sequences were obtained: 3D T1 weighted, T2 FLAIR, T2 weighted, diffusion weighted imaging (DWI, b-0, b-1000), SWAN (Susceptibility weighted Angiography), 3D Time of Flight MR angiography (3D-TOF-MRA). CW anatomy of each individual was evaluated using both 3DTOF MRA MIP reconstructions and source images.

The duplex ultrasonographic examination of extracranial arteries (vertebral and carotid) was performed by using the 7.5 MHz linear array transducer of Aloka Prosound F 75 ultrasound system. The diameter of VA in our previous study was measured similarly [7].

### Image analysis

MRA were reviewed by two independent neuroradiologists. If they had disagreements regarding the configuration of the circle of Willis, they discussed it until a consensus was reached.

The classification of CW and VA was carried out as follows:

1. When interpreting MRA, the presence or absence of PCcoA and P1 segment of PCA was assessed. P1 segment and the PCoA were scored as normal (diameter ≥0.8 mm), hypoplastic (diameter <0.8 mm in MRA), absent or non-visualised. The threshold of 0.8 mm in MRA was chosen in order to be consistent with other studies reported in literature [8].
2. The posterior part of the circle of Willis was defined as complete in cases of presence of both PCoA and P1 segment of PCA with diameter ≥0.8 mm. All other variants were defined as the incomplete posterior part of CW.
3. We defined the circle of Willis as fetal if PCA arises from the internal carotid artery, independent on the presence or absence of the atretic P1 segment. The fetal configuration is the variant in which the diameter of P1 segment of PCA is smaller than the diameter of PCoA. The vessels stems from ICA that had a diameter equal to or smaller than the diameter of the precommunicating segment P1 of the PCA were classified as PCoA. In cases of adult configuration, P1 segment of PCA has a diameter larger than PCoA while in transitional configuration, P1 segment and PCoA have close diameters. The subjects with adult and rarely found transitional CW configuration were included in the same group. This group was named as “adult “type CW.
4. The posterior part of CW was documented as presence of PCoA, absence/hypoplastic PCoA and fetal PCA (of FCW). The subjects with hypoplastic PCoA were included in the same group as those with absence of PComA since both groups have minimal or no possibilities to compensate the reduced posterior circulation from carotid arteries through PCoA in comparison with the individuals with presence of normal PCoA.
5. The absence of A1 segment of the anterior cerebral artery (ACA) was documented. In these cases both A2 segments are supplied by the existing A1 from the contralateral ACA.
6. VAH was established according to MRA (V4 segment) and duplex scanning (V1-V3 segment). We defined VA as hypoplastic when VA diameter in the entire course was less than 2.5 mm or an asymmetry ratio was equal or greater than 1:1.7. The definition was based on the findings obtained by authors [9], [10], [11] about the reduced flow velocities or higher peripheral resistance in subjects with the VA diameter <2.5 mm. We also studied the group named “VAH-PICA” with VA aplasia or hypoplastic VA not communicating with BA and terminating in PICA or the neck, i.e. subjects with the possibility of the greatest reduced blood flow through VA. The differential features of hypoplastic VA include a smooth narrowing of the lumen, straight walls while atherosclerosis presents with irregular walls and an uneven narrowing of the lumen with a more or less sharp angulation of filling defects.

We present a pattern of the posterior part of CW found in our individuals. We have estimated what concomitant vascular variants of the vertebrobasilar system are more common in FCW in comparison with other variants of CW. We assessed if the posterior part of CW differs in subjects with normal VA, VAH communicating with BA and VAH not communicating with BA or VA aplasia. We present the association between the anterior part of CW (absence of A1 segment) as an additional interesting finding which supports the hypothesis that the development of vessels in the anterior and posterior parts may be dependent.

### Statistics

The *Chi* square independence (χ2) test was applied in carrying out the comparison between the categorical variables, while Fisher’s exact test was used in the case of a small sample size. For multiple comparisons the Bonferroni adjustment was made. Continuous variables meeting the assumptions of normality were analysed using t-tests for independent groups. The chosen significance level was α = 0.05.

### Ethics

This study was approved by the Ethics Committee for the Vilnius region (No. 158200-15-767-281.). All the patients signed a written informed consent that allowed the researchers to use the data from their medical records.

## Results

### Posterior part of CW

The characteristics of the posterior part of CW are presented in Table 1. FCW was found in 15.9% of subjects free of stroke and TIA. Side-related differences in the posterior part of CW observed in both types of CW did not reach a statistically significant difference. In 47.9% of individuals both PCoA were absent or hypoplastic. 2.3% of the subjects had both-sided FCW.

**Table 1:** The characteristics of the posterior part of the circle of Willis (n = 923).

Type of posterior part of CW	N	Proportion (%)
Adult type	776	84.1
• Absence of both PCoA	442	47.9
• Absence of one PCoA	191	20.7
◦ Absence of left PCoA	112	12.1
◦ Absence of right PCoA	79	8.6
• Presence of both PCoA	143	15.5
Fetal type	147	15.9
Side of fetal circle of Willis		
• Left-sided	58	6.3
• Right-sided	68	7.4
• Both-sided	21	2.3
Contralateral PCoA		
• Absence/hypoplasia	81	8.8
• Normal	45	4.9
• Fetal circle of Willis (both- sided)	21	2.3

PCoA-posterior communicating artery; CW—circle of Willis.

### Demographic characteristics and coexisting arterial variants in adult CW and FCW (Table 2)

The proportion of men and women did not differ in both configurations of CW. The coexisting VAH was more common in subjects with FCW than in subjects with adult CW (correspondingly, 28.6% and 13.4%, p<0.001). Aplasia of A1was rare in both groups, although aplasia of A1 was more common in the group with FCW compared to those with adult CW. Moreover, in the majority of the subjects with FCW (in 6 of 7 cases), A1 aplasia was found ipsilaterally to FCW. Otherwise, the larger proportion of aplasia of A1 in the group with FCW compared to those without FCW was due to aplasia of A1 in ipsilateral to fetal PCA side. In these subjects, the carotid artery is supplying blood to PCA and MCA, while ACA is receiving blood from the contralateral carotid artery.

**Table 2:** The comparison of coexisting characteristics in FCW and adult CW.

	Fetal circle of Willis N = 147	Adult circle of Willis N = 776	p-value
Demographics:			
Men	79 (53.7%)	448 (57.7%)	NS*
Age	46.4±1.4	48.1±0.4	NS
Vertebrobasilar system			
VAH	42 (28.6%)	104 (13.4%)	<0.001
VAH terminating in PICA/aplasia	16 (10.9%)	16 (2.1%)	<0.001
BA hypoplasia /aplasia	2 (1.4%)	5 (0.64%)	NS
Fenestretion of BA	1 (0.7%)	3 (0.4%)	NS
Anterior part: Aplasia of A1 (ACA artery) segment and both A2 segments are supplied from one side by the existing A1	7 (4.8%) Ipsilateral side: 6 (4.1%) Contralateral side: 1 (0.7%)	13 (1.7%)	0.018

VAH-vertebral artery hypoplasia; BA-basilar artery; PICA-posterior inferior cerebellar artery; ACA-anterior cerebral artery; CW-circle of Willis. NS-p>0.05

### Association between VAH and the pattern of the posterior part of CW

The association between VAH and the variants of the posterior part of CW is presented in Table 3. The pattern of the posterior part of CW in subjects with VAH differs from those with normal VA. FCW was more frequent in individuals with VAH than in those with normal VA (accordingly, 28.8% vs. 13.5%, p<0.001), while the absence/hypoplasia of both PCoA was more common in subjects with normal VA in comparison to those with VAH (accordingly, 50.7% and 32.9%, p<0.001).

**Table 3:** The comparison of the posterior part of CW in patients with VAH and normal VA.

	Normal (n-777)	VAH (n = 146)	P-value
Adult type:			
Absence/hypoplasia of both PCoA	394 (50.7%)	48 (32.9%)	<0.001
Absence/hypoplasia of one PCoA	164 (21.1%)	27 (18.5%)	NS*
Presence of both PCoA	114 (14.7%)	29 (19.9%)	NS
Fetal circle of Willis	105 (13.5%)	42 (28.8%)	0.001
Unilateral	96 (12.4%)	30 (20.5%)	0.008
Bilateral	9 (1.2%)	12 (8.2%)	<0.001

VAH-vertebral artery hypoplasia; VA-vertebral artery; PCoA-posterior communicating artery; CW-circle of Willis. NS-p>0.05

### Difference of the posterior part of CW in subjects with VAH not communicating with the basilar artery and those with VAH communicating with the basilar artery

The above mentioned regularity was even more striking in the least developed hypoplastic VA that do not communicate with the basilar artery (Table 4). Half of the patients with VAH—PICA had FCW compared to 13.5% of those with normal VA diameter and to 22.8% of individuals with VAH that communicates with the basilar artery, p<0.005. Moreover, the proportion of bilateral FCW was largest in the subjects with VAH-PICA. On the contrary, the absence of both PCoA was most frequent in the group with normal VA and rare in VAH-PICA group (accordingly, 50.7% and 12.5%, p<0.001). Bonferroni adjustment did not impact the statistical significance.

**Table 4:** Comparison of the posterior part of CW in patients with a different degree of VAH.

	Normal (n = 777)	VAH-BA (n = 114)	VAH-PICA (n = 32)	p-value
Adult type:				
Absence/hypoplasia of both PCoA	394 (50.7%)	44 (38.6%)	4 (12.5%)	0.001
Absence/hypoplasia of one PCoA	164 (21.1%)	22 (19.3%)	5 (15.6%)	NS
Presence of both PCoA	114 (14.7%)	22 (19.3%)	7 (21.9%)	NS
Fetal circle of Willis	105 (13.5%)	26 (22.8%)	16 (50%)	0.001
Unilateral	96 (12.4%)	20 (17.5%)	10 (31.3%)	0.004
Bilateral	9 (1.2%)	6 (5.3%)	6 (18.8%)	0.001

VAH-vertebral artery hypoplasia; PCoA-posterior communicating artery; CW-circle of Willis; VAH-BA- hypoplastic vertebral artery communicating with the basilar artery; VAH-PICA- hypoplastic vertebral artery not communicating with the basilar artery and terminating in the posterior inferior cerebellar artery (PICA), neck or aplasia NS-p>0.05.

### The sides of VAH and FCW

VAH and FCW were more frequently observed on the same side: VAH was observed in ipsilateral to fetal PCA side in 78.6% of cases, VAH—PICA–in ipsilateral to fetal PCA side in 87.5% of cases.

## Discussion

FCW was found in 15.9% of individuals, bilaterally—in 2.3% of cases. According to other authors, the proportion of FCW ranges from 11 to 32% [1], [3], [12], [13], [14]. VAH was observed in 12.5% of subjects. According to the data presented by other authors, depending on the VAH definition, the method of examination and the category of population, this proportion ranges from 1.9% to 25% [9]. Therefore, the population under our investigation was a typical population.

FCW was more frequently observed in subjects with VAH, i.e. with an insufficiently developed proximal part of the vertebrobasilar system, compared to those with normal VA diameter. Among individuals with a very small VA not communicating with the basilar artery compared to those subjects whose VA is wider and forms the basilar artery, the proportion of FCW was larger. Moreover, FCW was more common in ipsilateral to VAH side rather than contralateral.

According to [6], BA diameter is inversely associated with FCW. Otherwise, FCW was more common in individuals with an insufficiently developed distal part of the vertebrobasilar system which can lead to inadequate posterior circulation, rather than in those with the normal basilar artery. The influence of an insufficiently developed proximal part of the vertebrobasilar system, including VAH or aplasia on the posterior circulation insufficiency and as a consequence the demand to compensate the possible inadequate blood supply to the brain is under discussion. The hypothesis that VAH can lead to the posterior circulation insufficiency is supported by our results that with the decreasing VA diameter, the risk of stroke/TIA increases [7]. Many authors estimate VAH as an independent predictor of stroke or TIA [9]. Moreover, VAH can lead to a relative regional hypoperfusion in the PICA territory [15].

The limitation of our study is the fact that we have examined only adult population. Although our data support the hypothesis about association between small vertebral arteries and the posterior part of the circle of Willis, we lack data about the vessels development in the fetal period and in different periods of postnatal life (infants, children, adults). One of the possible explanations about the cause of the association between the posterior part of CW and the VA size is the well known facts about the embryological development of brain vessels described by many authors [1], [2], [3], [4]. As described in above mentioned studies during the embryological development the anterior circulation supplies the occipital region, the brain stem and the cerebellum via multiple anastomoses because the posterior circulation is not yet well developed. After the development of VA and sufficient posterior circulation, these anastomoses regress. FCW as a result of failed regression that may be associated with insufficient blood supply via the insufficiently developed hypoplastic vertebrobasilar system. In these cases, the carotid artery may particularly recall the role of the vertebrobasilar system by supplying blood to the posterior fossa as in the embryological development. The greater proportion of FCW in subjects with more severe VAH whose blood flow through VA is reduced to a greater degree supports the hypothesis that with the decreasing blood supply from VA to the brain, the possible inadequate perfusion in posterior circulation is more frequently compensated through FCW from anterior circulation. In case of small diameter VA, compared to normal diameter VA, FCW may provide better blood supply to the brain and prevent from cerebral ischemia. To support or to reject this hypothesis, the future investigations about brain vessels in different postnatal and embryological periods are needed.

In summary, the pattern of the posterior part of CW in stroke/TIA-free subjects with VAH and normal VA was different. The proportion of absence/hypoplasia of both PCoA, i.e. the absence /hypoplasia of primary collaterals was larger in subjects with normal proximal circulation, i.e. normal VA diameter compared to those with insufficiently developed proximal part of the vertebrobasilar system, VAH. And vice versa, the proportion of FCW was larger in those with VAH compared to those with normal VA diameter. The proportion of subjects with a complete posterior part was larger in those with VAH although the difference did not reach a statistically significant difference. These results support the hypothesis that in cases of small vertebral arteries the collateral circulation through PCoA or FCW may be important for prevention of stroke/TIA in the posterior circulation.

Future investigations are needed in order to assess whether in cases of VAH the configuration of the posterior part of CW can prevent or increase the stroke/TIA risk. The study [1] revealed that the coexistence of the basilar artery hypoplasia, VAH and the fetal CW were more common in stroke patients. However, in the above mentioned study, the role of FCW is not clear. Is FCW an independent stroke predictor, or is FCW not able to compensate the reduced blood flow in cases of coexistence of small proximal and distal parts of the vertebrobasilar system? Future investigations are needed on the associations between a small vertebrobasilar system, CW configuration and neurological symptoms such as vertigo.

We did not analyze the anterior part of CW in this manuscript. However we present the association between the anterior part of CW (absence of A1 segment) as an additional interesting finding which supports the hypothesis that the development of vessels in the anterior and posterior parts may be dependent. In cases of FCW, the territory supplied with blood by the carotid artery increases up to three arteries (ACA, middle cerebral artery (MCA) and PCA). A1 aplasia in most cases is found ipsilaterally to fetal PCA and may be associated with the need to redistribute the blood flow in the anterior circulation and to reduce the territory of blood supply from the carotid artery from three arteries territory (ACA, MCA, PCA) to two arteries territory (MCA, PCA, while both ACA are supplying from the contralateral carotid artery.

## Conclusions

Individuals with VAH have a different pattern of the posterior part of CW in comparison with those with normal VA diameter. With the increasing degree of VAH, the proportion of FCW increases while the proportion of absence/hypoplasia of both PCoA decreauses.

## Supporting information

S1 Data Basic data 1. (SAV)

S2 Data Basic data 2. (XLS)
